# Incorporating COVID-19 into Acute Febrile Illness Surveillance Systems, Belize, Kenya, Ethiopia, Peru, and Liberia, 2020–2021

**DOI:** 10.3201/eid2813.220898

**Published:** 2022-12

**Authors:** David C. Shih, Rachel Silver, Olga L. Henao, Aynalem Alemu, Allan Audi, Godfrey Bigogo, Josh M. Colston, Elijah P. Edu-Quansah, Timothy A. Erickson, Andargachew Gashu, G. Burgess Gbelee, Sarah M. Gunter, Margaret N. Kosek, Gorbee G. Logan, Joy M. Mackey, Adrianna Maliga, Russell Manzanero, Gerhaldine Morazan, Francis Morey, Flor M. Munoz, Kristy O. Murray, Thelma V. Nelson, Maribel Paredes Olortegui, Pablo Penataro Yori, Shannon E. Ronca, Francesca Schiaffino, Adamu Tayachew, Musse Tedasse, Mesfin Wossen, Denise R. Allen, Pawan Angra, Amanda Balish, Madeline Farron, Marta Guerra, Amy Herman-Roloff, Victoria J. Hicks, Elizabeth Hunsperger, Lilit Kazazian, Matt Mikoleit, Peninah Munyua, Patrick K. Munywoki, Angella Sandra Namwase, Clayton O. Onyango, Michael Park, Leonard F. Peruski, David E. Sugerman, Emily Zielinski Gutierrez, Adam L. Cohen

**Affiliations:** Centers for Disease Control and Prevention, Atlanta, Georgia, USA (D.C. Shih, R. Silver, O.L. Henao, D.R. Allen, P. Angra, A. Balish, M. Farron, M. Guerra, V.J. Hicks, L. Kazazian, M. Mikoleit, A.S. Namwase, M. Park, L.F. Peruski, D.E. Sugerman, A.L. Cohen);; Ethiopian Public Health Institute, Addis Ababa, Ethiopia (A. Alemu, A. Gashu, A. Tayachew, M. Tedasse, M. Wossen);; Kenya Medical Research Institute, Kisumu, Kenya (A. Audi, G. Bigogo);; University of Virginia, Charlottesville, Virginia, USA (J.M. Colston, M.N. Kosek, P. Penataro Yori, F. Schiaffino);; African Field Epidemiology Network, Monrovia, Liberia (E.P. Edu-Quansah);; Texas Children’s Hospital, Houston, Texas, USA (T.A. Erickson, S.M. Gunter, A. Maliga, F.M. Munoz, K.O. Murray, S.E. Ronca);; Baylor College of Medicine, Houston (T.A. Erickson, S.M. Gunter, J.M. Mackey, A. Maliga, F.M. Munoz, K.O. Murray, S.E. Ronca);; National Public Health Institute of Liberia, Monrovia (G.B. Gbelee, T.V. Nelson);; Ministry of Health, Monrovia (G.G. Logan);; Ministry of Health and Wellness, Belmopan, Belize (R. Manzanero, G. Morazan, F. Morey);; Asociación Benéfica Prisma, Iquitos, Peru (M. Paredes Olortegui);; US Centers for Disease Control and Prevention, Nairobi, Kenya (A. Herman-Roloff, E. Hunsperger, P. Munyua, P.K. Munywoki);; US Centers for Disease Control and Prevention, Kisumu, Kenya (C.O. Onyango);; US Centers for Disease Control and Prevention, Guatemala City, Guatemala (E. Zielinski Gutierrez)

**Keywords:** COVID-19, respiratory infections, severe acute respiratory syndrome coronavirus 2, SARS-CoV-2, SARS, coronavirus disease, zoonoses, viruses, coronavirus, fever, acute disease, sentinel surveillance, incidence, epidemiology, global health, Belize, Kenya, Ethiopia, Peru, Liberia

## Abstract

Existing acute febrile illness (AFI) surveillance systems can be leveraged to identify and characterize emerging pathogens, such as SARS-CoV-2, which causes COVID-19. The US Centers for Disease Control and Prevention collaborated with ministries of health and implementing partners in Belize, Ethiopia, Kenya, Liberia, and Peru to adapt AFI surveillance systems to generate COVID-19 response information. Staff at sentinel sites collected epidemiologic data from persons meeting AFI criteria and specimens for SARS-CoV-2 testing. A total of 5,501 patients with AFI were enrolled during March 2020–October 2021; >69% underwent SARS-CoV-2 testing. Percentage positivity for SARS-CoV-2 ranged from 4% (87/2,151, Kenya) to 19% (22/115, Ethiopia). We show SARS-CoV-2 testing was successfully integrated into AFI surveillance in 5 low- to middle-income countries to detect COVID-19 within AFI care-seeking populations. AFI surveillance systems can be used to build capacity to detect and respond to both emerging and endemic infectious disease threats.

Acute febrile illness (AFI) is a common clinical syndrome that can be caused by various pathogens, ranging from treatable and vaccine-preventable infectious agents to newly emerging pathogens with pandemic potential (*1*). AFI is characterized by recent onset of fever with or without localizing symptoms, and etiologies can vary depending on the population, region, season, or patient age. Comparable data describing the epidemiology and distribution of AFI across countries and regions are limited, particularly among low- and middle-income countries (*2*). In countries with limited laboratory diagnostic testing resources, common causes of fever are challenging to diagnose through clinical assessment alone when localizing symptoms are absent and endemic disease prevalence is unknown. Many low- and middle-income countries struggle to build needed laboratory diagnostic capacity because of resource constraints. Reduced diagnostic capability can lead to inaccurate empirical diagnosis and treatment of emerging infectious and other febrile diseases and encumber both the healthcare system and the population it serves. Management of febrile illness in a primary healthcare clinic can differ from that in a hospital setting in which empiric diagnosis and treatment can be crucial for patients with severe febrile illness or sepsis. Nevertheless, improved knowledge of locally circulating infectious disease etiologies can inform these diagnoses in both healthcare settings. Lack of knowledge of endemic etiologies for AFI can result in delayed diagnoses and treatment and overuse of antimicrobial drugs, which can undermine trust in healthcare systems and governments (*3*).

AFI surveillance is a critical component of a global health strategy and aims to generate data and build capacity to detect and respond to both emerging and endemic infectious disease threats (*4*,*5*). For example, AFI surveillance detected a chikungunya virus outbreak in Puerto Rico in 2014, and the first Zika virus infections in 50 years were identified in Uganda in 2017 through AFI surveillance (*6*,*7*). Through the collection and interpretation of epidemiologic and laboratory data, AFI surveillance data can provide estimates of the occurrence and distribution of disease, inform clinical care practices (including antimicrobial stewardship), and guide prevention measures and public health action. Furthermore, flexible AFI surveillance systems that can adapt to and be leveraged for pathogen-specific needs have been indispensable during the emergence of infectious disease threats, such as Zika virus in the Americas and French Polynesia, yellow fever and Ebola viruses in Africa, and now SARS-CoV-2 worldwide (*8*–*10*).

On March 11, 2020, the World Health Organization (WHO) declared COVID-19 a pandemic (*11*). In response, the US Centers for Disease Control and Prevention (CDC) developed guidance on adapting AFI surveillance systems to integrate SARS-CoV-2 testing into existing or planned AFI activities in various countries (*12*). CDC recommended maintaining the same selection criteria for patients that were used before surveillance integration, which enabled countries to incorporate AFI surveillance systems with minimal disruption. AFI surveillance could be vital for monitoring COVID-19, which can cause fever without localizing symptoms and evade influenza-like illness surveillance if no respiratory symptoms are present (*13*–*17*). We describe how AFI surveillance systems were leveraged to detect and characterize SARS-CoV-2 infections using preliminary data from 5 low- to middle-income countries that incorporated SARS-CoV-2 detection into their AFI surveillance programs.

## Materials and Methods

### General AFI Surveillance Methods

To select sentinel sites for AFI surveillance, CDC, host governments, and implementing partners considered various factors, including the presence of existing and adaptable data collection platforms, patient volume, known infectious disease hotspots or priority regions, laboratory infrastructure and specimen transport networks, geographic representation, and urban versus rural catchment areas. Surveillance staff members were trained in procedures used for patient screening, consent and enrollment, data collection, and specimen collection and transportation. Staff screened patients with acute fever or a history of acute fever in both outpatient and inpatient settings and enrolled patients who met the AFI case definition and consented to participate in surveillance activities. AFI case definitions were based on pathogen-specific priorities for each country or region. Staff members used questionnaires to collect demographic, clinical, and exposure data from enrolled patients. Epidemiologic data were linked to laboratory data either manually or automatically, depending on the country’s data management system, through a unique patient identifier.

Surveillance staff collected whole blood from participants in each country that implemented AFI surveillance. A TaqMan array card that detects multiple targets of both bacterial and viral pathogens from a single sample was developed specifically for AFI surveillance and has been successfully implemented (*18*). This array card, which uses a singleplex microfluidics multiple pathogen PCR detection system, was commonly used to test for pathogens in blood and is not commercially available. CDC partners often use custom versions according to the country’s pathogens of interest. In addition, singleplex reverse transcription PCR, multiplex PCR panels, point-of-care rapid testing, or serologic tests were used to identify specific viral or parasitic pathogens. Depending on the country’s protocol and pathogens under surveillance, additional specimens were collected, including respiratory specimens, such as nasopharyngeal, oropharyngeal, and nasal mid-turbinate swab samples, as well as saliva, urine, feces, or eschar samples. CDC and partners selected the list of pathogens for testing according to the pathogens of interest in each country or region, laboratory capabilities, and potential for developing surveillance and laboratory capacity in-country.

### COVID-19 Integration

In response to the COVID-19 pandemic, CDC collaborated with partners in different countries to incorporate COVID-19 surveillance into existing or planned AFI surveillance systems. CDC and implementing partners defined how surveillance would be performed and adapted laboratory testing algorithms and case selection criteria, if necessary, to account for respiratory symptoms. COVID-19–specific questions were incorporated into existing questionnaires to ascertain COVID-19–like symptoms, such as shortness of breath, loss of taste, and loss of smell, and COVID-19 vaccination status. Potential exposures were documented, including attendance at large gatherings, contact with anyone suspected of having or confirmed to have COVID-19 or a similar illness, or domestic travel 14 days before symptom occurrence. If respiratory specimens were not collected under the original AFI surveillance protocol, >1 specimen was obtained from all consenting patients with AFI.

### Country-Specific Methods

The 5 countries evaluated in this study were Belize, Kenya, Ethiopia, Peru, and Liberia. We analyzed AFI and COVID-19 surveillance methods for each country, aggregating AFI surveillance enrollment data and SARS-CoV-2 test results. Methods for AFI surveillance and COVID-19 integration activities varied by country (Table 1).

**Table 1:** Summary of methods used for COVID-19 incorporation into acute febrile illness surveillance systems in Belize, Kenya, Ethiopia, Peru, and Liberia, 2020–2021*

Category	Belize	Kenya†	Ethiopia	Peru	Liberia
Surveillance start dates					
AFI	2020 Jan	2006 Jan	2021 Feb	2021 Feb	2018 Dec
COVID-19 integration	2020 Mar	2020 May	2021 Feb	2021 Feb	2021 Apr
No. sites	11	2	4‡	5	2
Inclusion criteria					
Age	>60 d	All ages	>5 y	>10 y	>2 y (AFI), >5 y (COVID-19)
Documented body temperature or history of fever	Axillary, oral, or rectal T >38°C or new fever <7 d before exam	Axillary T >38°C and <5 d of acute fever	Axillary, oral, or rectal T >38°C and fever for 2–14 d before exam	Axillary, oral, or rectal T >38°C and new fever <14 d before exam	Axillary, oral, or rectal T >37.5°C or fever <7 d before exam
Afebrile patients	>2 respiratory symptoms and high risk for or suspected SARS-CoV-2 infection or >2 GI symptoms	None	None	None	None
Exclusion criteria					
Surveillance protocol procedures	Previously enrolled within the past 7 d or declined follow up for disease outcomes	Previously enrolled	Previously enrolled	None	Previously enrolled within past year
Chief complaint on arrival or during hospitalization	Injury, trauma, or known cause of fever; returning with known cause of fever	Injury or trauma	Injury, trauma, focal infection, localizing symptoms, obstetric- or surgery-related cases	Focal infection or fever onset >24 h after hospitalization (inpatients only)	Injury, trauma, focal infection, returning with known cause of fever
Data use methods§					
Collection	REDCap and paper-based form	Windows-based platform	Paper-based form	REDCap	Paper-based form
Management	REDCap	Microsoft SQL servers	Microsoft Excel	Microsoft Access	Epi Info
Specimens	Blood, NP/OP swabs, feces, eschar swabs	Blood, NP/OP swabs;¶ urine	Blood, NP/OP swabs¶	Blood, nasal MT swabs, saliva	Blood, NP swabs¶
COVID-19 testing methods	Singleplex RT-PCR,# BioFire FilmArray respiratory panel**	RT-PCR#	Singleplex PCR#	CDC COVID-19 assay#††	TaqPath COVID-19 CE-IVD RT-PCR#‡‡

*Data are sorted by COVID-19 integration month. AFI, acute febrile illness; GI, gastrointestinal; MT, mid-turbinate; NP, nasopharyngeal; OP, oropharyngeal; RT-PCR, reverse transcription PCR; T, temperature. †Data are from Kenya’s population-based infectious disease surveillance sites with survey-defined catchment areas. ‡Of 5 designated sites, only 4 were operational because of security issues. §REDCap (https://www.project-redcap.org); Microsoft Excel, Access, SQL Server, and Windows-based platform (https://www.microsoft.com); Epi Info (https://www.cdc.gov/epiinfo). ¶Additional specimens collected after COVID-19 surveillance integration into regular AFI surveillance activities. #Tests performed specifically for SARS-CoV-2. **BioFire (https://www.biofiredx.com). ††2019 nCoV Real-Time RT-PCR Diagnostic Panel, Centers for Disease Control and Prevention (https://www.cdc.gov/coronavirus/2019-ncov/lab/testing.html). ‡‡TaqPath COVID-19 CE-IVD RT-PCR kit, Thermo Fisher Scientific (https://www.thermofisher.com).

Each country implemented sentinel surveillance (Table 2). AFI surveillance in Kenya took place specifically at 2 population-based clinics that were essentially sentinel sites but had well-defined catchment areas (*19*,*20*). An inclusion criterion for participation in the AFI surveillance system was a minimum body temperature of 38°C in each country except Liberia, which required a minimum body temperature of 37.5°C. Another inclusion criterion was a history of fever within a set number of days that was either combined with or instead of the minimum required body temperature. Belize was the only country that included afebrile patients if they had >2 respiratory symptoms, a history of travel, or other COVID-19 risk factors, or >2 gastrointestinal symptoms. All countries except Kenya had an age requirement for participants.

**Table 2:** Surveillance sites for COVID-19 incorporation into acute febrile illness surveillance systems in Belize, Kenya, Ethiopia, Peru, and Liberia, 2020–2021

Category	Belize	Kenya	Ethiopia	Peru	Liberia
City, no. hospitals	Belize City, 3; Corazal,1; Belmopan,1; Orange Walk,1; San Ignacio,1; Dangringa,1; Punta Gorda,1	None	Addis Ababa, 1; Harar, 1; Gonder, 1; Jimma, 1	Iquitos, 2	Monrovia, 1
City, no. clinics	San Pedro, 1; Independence,1	Asembo, 1; Nairobi, 1	None	Iquitos, 4; Mazan, 1	Monrovia, 1

Surveillance site staff collected epidemiologic data by using a combination of electronic and paper-based data collection tools and methods. Platforms, such as REDCap (https://www.project-redcap.org), Epi Info (https://www.cdc.gov/epiinfo), Microsoft Excel and Access (https://www.microsoft.com), or country-specific patient care systems were used for data entry and management. Laboratory staff tested all respiratory specimens collected from consenting participants for SARS-CoV-2 using PCR methods. Liberia was the only country to require a separate verbal agreement for SARS-CoV-2 testing.

Survey activities underwent human subjects review and received approval within their respective countries or institutions. AFI activities also underwent human subjects ethics review by CDC and were conducted in accordance with applicable CDC policy and federal law, including the code of federal regulations (CFR) and US codes (USC) 45 CFR part 46, 21 CFR part 56; 42 USC §241(d); 5 USC §552a; 44 USC §3501 et seq.

For each country, we summarized the information obtained for enrolled AFI surveillance participants during the data collection period and stratified the data by age and sex. CDC did not request or receive any personally identifiable data. The data collection period varied by country; the start date represents the month that COVID-19 surveillance was implemented, and the end date indicates when data were available for analysis in this study. Data collection in each country was ongoing as of June 3, 2022. We calculated the number and percentage of enrolled persons who were tested for SARS-CoV-2; the numbers and percentage of SARS-CoV-2–positive samples were calculated and stratified by age of participants. We used Microsoft Excel version 2102 for all calculations.

## Results

The data collection periods in the 5 countries ranged from 4 to 17 months (Table 3). Belize integrated SARS-CoV-2 testing in March 2020, Kenya in May 2020, Ethiopia in February 2021, Peru in February 2021, and Liberia in April 2021. A total of 5,501 patients with AFI were enrolled during the period from initiation of COVID-19 surveillance activities to when data were available for this analysis. Participants who were 15–44 years of age comprised 50% (817/1,627) of enrollees in Belize, 44% (51/115) in Ethiopia, and 66% (228/344) in Peru, whereas 81% (2,507/3,113) of enrolled patients in Kenya and 47% (141/302) in Liberia were <15 years of age. The sex distribution of participants was approximately equal in Belize (48% male patients, 788/1,627), Kenya (48% male patients, 1,487/3,113), and Peru (52% male patients, 178/344), whereas 43% (131/302) of participants in Liberia were male. In Ethiopia, 57% (65/115) of enrolled patients were male; however, 17% (20/115) of participants in Ethiopia had missing age and sex data.

**Table 3:** Demographic characteristics of surveillance participants and SARS-CoV-2 testing results after COVID-19 incorporation into acute febrile illness surveillance systems in Belize, Kenya, Ethiopia, Peru, and Liberia, 2020–2021*

Variables	Belize	Kenya	Ethiopia	Peru	Liberia
Data collection period	2020 Mar–2021 Jul	2020 May–2021 Sep	2021 Feb–Aug	2021 Feb–Oct	2021 Apr–Jul
Total no. enrolled patients	1,627	3,113	115	344	302
Sex†					
M	788 (48)	1,487 (48)	65 (57)	178 (52)	131 (43)
F	839 (52)	1,626 (52)	30 (26)	166 (48)	171 (57)
Unknown sex	0	0	20 (17)	0	0
Age groups, y†					
<5–14	473 (29)	2,507 (81)	2 (2)	9 (3)	141 (47)
15–44	817 (50)	502 (16)	51 (44)	228 (66)	113 (37)
45–64	231 (14)	75 (2)	22 (19)	81 (24)	41 (14)
>65	106 (7)	29 (1)	20 (17)	26 (8)	7 (2)
Unknown age	0	0	20 (17)	0	0
Tested for SARS-CoV-2, y‡					
<5–14	349 (74)	1,734 (69)	2 (100)	9 (100)	90 (64)
15–44	709 (87)	341 (68)	51 (100)	218 (96)	85 (75)
45–64	207 (90)	56 (75)	22 (100)	81 (100)	33 (80)
>65	97 (92)	20 (69)	20 (100)	26 (100)	7 (100)
Unknown age	0	0	20 (100)	0	0
Total	1,362 (84)	2,151 (69)	115 (100)	334 (97)	215 (71)
SARS-CoV-2 positive, y§					
<5–14	18 (5)	45 (3)	0	0	9 (10)
15–44	77 (11)	31 (9)	5 (10)	27 (12)	9 (11)
45–64	38 (18)	8 (14)	6 (27)	16 (20)	6 (18)
>65	18 (19)	3 (15)	8 (40)	8 (31)	1 (14)
Unknown age	0	0	3 (15)	0	0
Total	151 (11)	87 (4)	22 (19)	51 (15)	25 (12)

*Participants were enrolled during the indicated periods and sorted by the month data collection began. AFI, acute febrile illness. †No. (%) participants out of total enrolled. ‡No. (%) enrolled participants who were tested for SARS-CoV-2 in each age group. §No. (%) tested participants with positive SARS-CoV-2 samples in each age group.

The percentage of enrolled patients who were tested for SARS-CoV-2 was 84% (1,362/1,627) in Belize, 69% (2,151/3,113) in Kenya, 100% (115/115) in Ethiopia, 97% (334/344) in Peru, and 71% (215/302) in Liberia. Within each age group, >50% of enrolled participants consented to respiratory specimen collection and SARS-CoV-2 testing (Table 3). SARS-CoV-2 percent positivity varied by country. COVID-19 surveillance was integrated with AFI surveillance in early 2020 in Kenya and Belize. Among SARS-CoV-2–tested patients with AFI, samples from 4% (87/2,151) of patients in Kenya and 11% (151/1,362) in Belize were positive for the virus. COVID-19 integration began in early 2021 in Ethiopia, Peru, and Liberia. Among SARS-CoV-2 tested patients with AFI, samples from 19% (22/115) in Ethiopia, 15% (51/334) in Peru, and 12% (25/215) in Liberia were positive for SARS-CoV-2. Participants >65 years of age in Belize, Kenya, Ethiopia, and Peru had the highest percentage of SARS-CoV-2 positivity; 19% (18/97) of patients in Belize, 15% (3/20) in Kenya, 40% (8/20) in Ethiopia, and 31% (8/26) in Peru were SARS-CoV-2–positive in this age group. Participants 45–64 years of age had the second highest percentage of SARS-CoV-2 positivity: 18% (38/207) in Belize, 14% (8/56) in Kenya, 27% (6/22) in Ethiopia, and 20% (16/81) in Peru. In Liberia, participants 45–64 years of age had the highest (18% [6/33]) SARS-CoV-2 positivity, and patients >65 years of age had the second highest rate, 14% (1/7). In 4 countries, samples from male patients tested positive for SARS-CoV-2 more frequently than did samples from female patients: Belize, 13% (79/632) male patients versus 10% (72/730) female patients; Ethiopia, 25% (16/65) male patients versus 10% (3/30) female patients; Liberia, 13% (12/95) male patients versus 11% (13/120) female patients; and Peru, 20% (35/173) male patients versus 10% (16/161) female patients. In Kenya, samples from ≈4% (46/1,068) male patients and ≈4% (41/1,083) female patients tested positive for SARS-CoV-2.

## Discussion

AFI surveillance activities were successfully leveraged for the COVID-19 pandemic in Belize, Kenya, Ethiopia, Peru, and Liberia through the collection of relevant laboratory and epidemiologic data that could then be used to inform each country’s response to the disease. Developing a new surveillance system, particularly in a low- to middle-income country, takes a substantial amount of time, planning, resources, and personnel. However, including COVID-19 in planned or existing AFI surveillance systems resulted in an efficient response to an urgent need and increased the ability to build capacity for long-term disease surveillance. Belize and Kenya had existing AFI surveillance systems and were able to rapidly integrate COVID-19 into these systems. Belize integrated COVID-19 within 1 month and Kenya within 2 months after the March 2020 COVID-19 pandemic announcement by WHO. Peru and Ethiopia integrated COVID-19 surveillance during the launch of their AFI surveillance activities in February 2021, and Liberia implemented COVID-19 surveillance in April 2021.

The broad-spectrum AFI syndromic surveillance system complements pathogen-specific surveillance systems. AFI surveillance generally requires participants to have only an acute fever for inclusion, which then allows the detection of a wide variety of pathogens and COVID-19 cases with various clinical manifestations. SARS-CoV-2 infections that were detected through AFI surveillance might have potentially gone undetected if respiratory disease–specific surveillance had been the sole source of case findings.

Our results demonstrate that AFI surveillance can be adapted and leveraged for pandemic monitoring through established laboratory and reporting mechanisms. We found surge capacity testing for SARS-CoV-2 was successful by using existing AFI surveillance specimen collection and testing methods, which was demonstrated by the >69% of enrolled AFI participants tested for SARS-CoV-2 in each country. In addition, established AFI surveillance methods enabled collection of descriptive data for participants with COVID-19, including demographic information, potential exposures, and vaccine history. These data could be used to characterize the care-seeking, febrile population affected by COVID-19 in a specific country. Furthermore, the relationships and communication channels that were already established for reporting AFI epidemiologic and laboratory data to public health authorities in each country were used for submission of COVID-19 case data. These data informed case investigations, case management, or contact tracing efforts and contributed to situational awareness and general pandemic tracking. For example, Liberia’s COVID-19 cases detected through AFI surveillance were integrated into the country’s incident management system and enabled the Montserrado County health team to investigate and manage these cases. The surveillance teams in Kenya routinely shared confirmed case data with county Ministry of Health teams to assist appropriate responses, such as contact tracing, and provided reports and updates to the Ministry of Health and other parties tracking the pandemic. In addition, authorities in Belize used their AFI surveillance data on COVID-19 cases to inform and assist contact tracing efforts.

The WHO COVID-19 Detailed Surveillance Data Dashboard (*21*) shows COVID-19 case, death, and vaccination data reported worldwide through official communications and is supplemented with official data taken from ministry of health websites of different countries (*22*). We aimed to compare the test positivity rates from the WHO COVID-19 dashboard with the SARS-CoV-2 percent positivity in the AFI surveillance populations reported in this study. However, because of a lack of test volume data for some relevant weeks, we were only able to compare these statistics for Ethiopia. We divided the total number of COVID-19 cases reported on the dashboard for Ethiopia by the total number of persons tested for SARS-CoV-2 during February–August 2021 (Ethiopia’s AFI data collection time frame). The national test positivity rate reported by the WHO dashboard was 12%, which was below the 19% found in the AFI surveillance time frame. This difference is consistent with the types of populations that were surveyed. Most AFI surveillance participants described in this study were from a care-seeking population with acute symptomatic illness, which potentially yielded a higher proportion of SARS-CoV-2–positive samples. Hospitalized patients likely had more serious symptoms and a higher probability of SARS-CoV-2 infection than patients in outpatient clinics (*16*,*17*). Other factors, such as the level of community transmission and access to care, can also influence the percent positivity. As the COVID-19 pandemic evolves, the percentage of positive cases is expected to change depending on circulating variants, levels of immunity, and vaccination status in different communities.

Surveillance staff reported logistical and administrative challenges that affected their surveillance activities. Staff in Ethiopia encountered unexpected funding constraints and procurement issues that negatively affected sample collection supplies and limited AFI surveillance expansion to additional sites and testing for additional pathogens. Staff in Belize, Peru, and Liberia experienced shortages of nasopharyngeal swabs. Staff in Liberia borrowed swabs from the national reference laboratory, whereas surveillance staff in Peru switched to nasal mid-turbinate swabs. Peru experienced widespread nosocomial SARS-CoV-2 transmission, leading to treatment deferment for many patients with mild and moderate disease severity. Belize encountered a substantial decrease in participant enrollment in their AFI surveillance throughout all 11 healthcare facilities because of a strict government lockdown at the beginning of the pandemic. In addition, Belize, Kenya, and Peru reported issues with procuring personal protective equipment for use by facility staff.

The first limitation of our study is that harmonizing data from projects with slightly different methods created some challenges. Differences in inclusion and exclusion criteria and laboratory testing platforms made inter-country comparisons difficult; however, local circumstances and testing capacity often made these differences unavoidable. Furthermore, different conditions in each country made it impractical to restrict data to a specific period; thus, we showed all available data. Second, health facility–based sentinel surveillance was used rather than population-based surveillance, which limited the findings to the healthcare-seeking population. However, implementers selected sentinel sites that were broadly representative of their country’s care-seeking population. For example, Belize used most of the nation’s clinical sites, which comprehensively captured a high proportion of their care-seeking population. Third, sex and age data were missing in some cases, limiting the interpretation of some findings. In Ethiopia, sex and age data were missing for 17% of enrollees, although project staff were still able to estimate overall SARS-CoV-2 percentage positivity because 100% of participants consented to SARS-CoV-2 testing. Last, some enrolled patients might have had asymptomatic SARS-CoV-2 infection concurrent with another febrile illness, although this possibility is unlikely.

Molecular SARS-CoV-2 testing and genomic sequencing methods have promoted ongoing surveillance of COVID-19. In Peru, Belize, and Kenya, genomic sequencing is being used to track SARS-CoV-2 variants. Collection of COVID-19 data through AFI surveillance continues to evolve in all 5 countries included in our study. Those data offer possibilities for analyses of single-site trends, incorporation of additional testing methods (such as SARS-CoV-2 serologic tests), and identification of emerging variants and co-infections. Other descriptive and statistical analyses can also be performed by using demographic, clinical, epidemiologic, and laboratory testing data.

In conclusion, through examination of preliminary data from Belize, Ethiopia, Kenya, Liberia, and Peru, we have shown that SARS-CoV-2 testing can be integrated successfully into AFI surveillance systems. We reported SARS-CoV-2 percent positivity data among care-seeking AFI surveillance populations and demonstrated the utility of leveraging existing AFI surveillance systems for COVID-19 pandemic responses or pathogen-specific needs. Integrating pathogens, such as SARS-CoV-2, into existing surveillance systems builds capacity to prevent, detect, and respond to both emerging and endemic infectious disease threats in low- to middle-income countries.
